# Effect of furosemide deresuscitation on fluid distribution and bioelectrical impedance analysis parameters in septic patients: a secondary analysis of a double-blind, randomized controlled trial

**DOI:** 10.62838/jccm-2026-0051

**Published:** 2026-10-30

**Authors:** Dita Aditianingsih, Jefferson Hidayat, Anne Suwan Djaja, Theodorus Samuel Rahardja

**Affiliations:** Critical Care Division, Anesthesiology and Intensive Care Department, Faculty of Medicine Universitas Indonesia, Cipto Mangunkusumo National General Hospital, Jakarta, Indonesia

**Keywords:** Bioelectrical impedance analysis, Fluid distribution, Furosemide deresuscitation, Sepsis

## Abstract

**Introduction:**

Sepsis is a leading cause of mortality globally, accounting for 11 million deaths annually, particularly in ICU patients. Fluid therapy is critical in sepsis management, yet aggressive resuscitation often leads to fluid overload, worsening outcomes. Bioelectrical impedance analysis (BIA) is a noninvasive method for monitoring body fluid distribution, providing insights into fluid imbalances associated with sepsis mortality.

**Aim:**

This study evaluates the impact of furosemide-induced fluid deresuscitation on fluid distribution and clinical outcomes in patients with sepsis.

**Methods:**

A secondary analysis of a double-blind, randomized controlled trial was conducted in forty sepsis patients with cumulative fluid balance >1,500 mL and urine output <0.8 mL/kg/hour. Patients were randomised to the intervention (furosemide infusion) and control (placebo) groups. BIA was used to assess total body water (TBW), extra-cellular water (ECW), intracellular water (ICW), and related parameters at baseline and after 48 hours. Outcomes included changes in fluid distribution, ICU length of stay, RRT use, and 28-day mortality.

**Results:**

At baseline, no significant differences were observed in demographic or clinical characteristics between the groups. All BIA parameters showed no significant differences, except for the ECW/ICW ratio and ICW levels. After 48 hours, the intervention group exhibited a significant reduction in ECW/ICW ratio (P = 0.005) and ICW levels (P = 0.004), while the control group showed increases in ICW and TBW. Mortality rates were higher in the control group (36.8% vs 25.0%), though not statistically significant (P = 0.423). Severe hypoalbuminemia was more frequent in the control group, potentially contributing to fluid imbalance.

**Conclusions:**

While furosemide-induced deresuscitation may influence fluid redistribution, its effect on clinical outcomes remains inconclusive.

## Introduction

Sepsis is a global health threat with high incidence and mortality rates. In 2017, sepsis was responsible for 49 million cases and 11 million deaths, accounting for 19.7% of global mortality[1,2]. It remains a leading cause of death in hospitalized patients, particularly in ICUs[3]. Data from 2012 at Cipto Mangunkusumo Hospital (RSCM), Jakarta, showed 23 out of 84 ICU cases were sepsis or septic shock, with a mortality rate of 47.8%[4].

Sepsis triggers systemic inflammation, leading to endothelial damage, capillary leakage, and obstruction caused by leukostasis and thrombosis. Capillary leakage begins early, resulting in fluid accumulation upon hospital admission. Fluid therapy is the primary treatment for hemodynamically unstable patients[5]. According to the Surviving Sepsis Campaign (SSC), initial management includes 30 mL/kg of crystalloid fluid if hypotension occurs or lactate levels exceed 4 mmol/L [6]. Although aggressive fluid therapy may reduce renal ischemia by diluting nephrotoxins and preventing tubular obstruction, it also exacerbates fluid overload, leading to organ swelling and compression, especially in encapsulated organs such as the kidneys [5,7].

Bioelectrical impedance analysis (BIA) is a reliable, noninvasive method for assessing body composition by transmitting low-level electrical currents through electrodes placed on the skin [8,9]. BIA provides critical information on body fluid distribution, including total body water (TBW), composed of extracellular water (ECW) and intracellular water (ICW). ECW, comprising one-third of TBW, includes interstitial fluid and plasma, while ICW constitutes two-thirds of TBW, representing intracellular fluid [10,11]. These fluid compartments are vital to resuscitation strategies.

In sepsis patients, aggressive fluid resuscitation increases TBW and ECW, with a higher ECW/TBW ratio linked to worse outcomes, including higher mortality[10]. Studies show non-survivors have significantly higher ECW relative to TBW during resuscitation, indicating extracellular edema and intracellular dehydration[12]. BIA has revealed significant changes in ECW/TBW and ICW/TBW ratios during therapy, with non-survivors exhibiting higher ECW/TBW and lower ICW/TBW ratios.[10]

High cumulative fluid balance is strongly associated with increased mortality[ 13, 14, 15]. Patients with a low cumulative fluid balance at ICU admission have less severe illness and better outcomes [14]. The RADAR-2 study confirmed the feasibility and safety of deresus-citation using furosemide, showing no significant adverse effects compared to standard care[16]. Similarly, Messmer et al. found no significant difference between patients undergoing active deresuscitation and those receiving standard care [17]. However, Li et al. reported that furosemide-induced deresuscitation delayed RRT and mechanical ventilation while reducing in-hospital mortality[18].

This study highlights the critical importance of fluid monitoring in sepsis patients, with particular focus on evaluating body fluid distribution during the deresus-citation phase to optimize treatment outcomes. Therefore, this randomized controlled trial aims to provide a detailed comparison of fluid distribution and BIA parameters between sepsis patients undergoing deresuscitation and those in the control group. By addressing this gap, the study seeks to contribute valuable insights into the role of fluid distribution monitoring in improving sepsis management strategies.

## Methods

This study is a secondary analysis of a previous study employing a double-blind, randomized clinical trial design to compare fluid distribution and bioelectrical impedance analysis (BIA) parameters in sepsis patients undergoing fluid deresuscitation with their controls. The study was conducted in the intensive care unit (ICU) at Cipto Mangunkusumo Hospital in Jakarta, Indonesia, from July to December 2023. No additional randomizations or interventions were performed on the previously established dataset

This study was conducted in accordance with the ethical principles outlined in the Declaration of Helsinki. Ethical approval was obtained from the Ethics Committee of the Faculty of Medicine, Universitas Indonesia (No. KET-746/UN2.F1/ETIK/PPM.00.02/2023, dated June 5th 2023) for the original trial. This study was registered on ClinicalTrials.gov (NCT05939245, dated June 25th, 2023). Written informed consent was procured from patients or their legal guardians after explaining the study’s objectives, benefits, procedures, and potential risks. Participation was voluntary, and confidentiality of participant data was strictly maintained.

The target population was iterations from the original study, consisting of sepsis patients treated in the ICU. Patients were eligible for inclusion if they had a cumulative fluid balance exceeding 1,500 mL and a urine output of less than 0.8 mL/kg/hour. Exclusion criteria included a history of kidney transplantation, stage V chronic kidney disease, valvular heart disease, or congenital heart disease. Patients were withdrawn from the study if they were anuric for more than 24 hours upon ICU admission or died within the first 48 hours of ICU admission.

A total of 40 sample numbers were generated using the Researcher Randomizer application. Subjects were randomly assigned to either the intervention or control group in a 1:1 ratio. Group allocation was conducted by a third party to maintain blinding. In the intervention group, subjects received a continuous infusion of furosemide at 2 mg/hour, titrated to achieve a urine output of 1–2 mL/kg/hour for 48 hours. In cases of hypotension (mean arterial pressure [MAP] < 65 mmHg), norepinephrine was administered at an initial dose of 0.05 mcg/kg/min, with fluid loading of 4 mL/kg body weight (or 300 mL) over 5–10 minutes to achieve a MAP ≥ 65 mmHg. In the control group, subjects received a continuous infusion of placebo (saline) at 2 mL/hour, titrated similarly to achieve the target urine output. Norepinephrine and fluid loading were administered for hypotension, following the same protocol as in the intervention group.

BIA data were collected at baseline and at hour 48. BioScan Touch-i8 (Maltron International Ltd., Essex, UK) was used to measure fluid accumulation and body composition parameters. ICU length of stay, RRT usage, and 28-day mortality were recorded as secondary outcomes. Adverse events, including significant electrolyte disturbances (e.g., severe hyponatremia, hypokalemia, and hypochloremia) or reduced urine output (<0.5 mL/kg/hour), were managed following ICU protocols. The intervention would be paused or discontinued if necessary, and patients requiring additional interventions, such as RRT, would continue to be monitored and included in the analysis.

The minimum sample sizes were calculated for categorical and numerical variables using standard samplesize formulas for unpaired comparative trials. Based on the calculations, the largest sample size requirement was for the mortality parameter, which required 18 subjects per group [14]. Allowing for a 10% dropout rate, the final total sample size was set at 40 subjects, with 20 subjects in each group. Data were analyzed using SPSS version 26.0. Descriptive statistics were used to summarize categorical variables as frequencies and percentages, and numerical variables as medians with interquartile ranges. Confidence intervals for medians were estimated using bootstrap resampling (1000 iterations). The 95% CI was defined as the 2.5th and 97.5th percentiles of the bootstrap distribution. Normality of data was assessed using the Shapiro-Wilk test. Bivariate analyses were performed using independent t-tests or Mann-Whitney U tests for numerical variables and chi-square or Fisher’s exact tests for categorical variables. A p-value of <0.05 was considered statistically significant.

## Results

This study included 40 patients who met the inclusion criteria. None were excluded before randomization. The 40 patients were randomized into intervention (n = 20) and control (n = 20) groups (Figure 1). One subject in the control group was excluded due to mortality within 48 hours of ICU admission, resulting in 19 subjects in the control group and 20 subjects in the intervention group for analysis.

**Figure 1.**
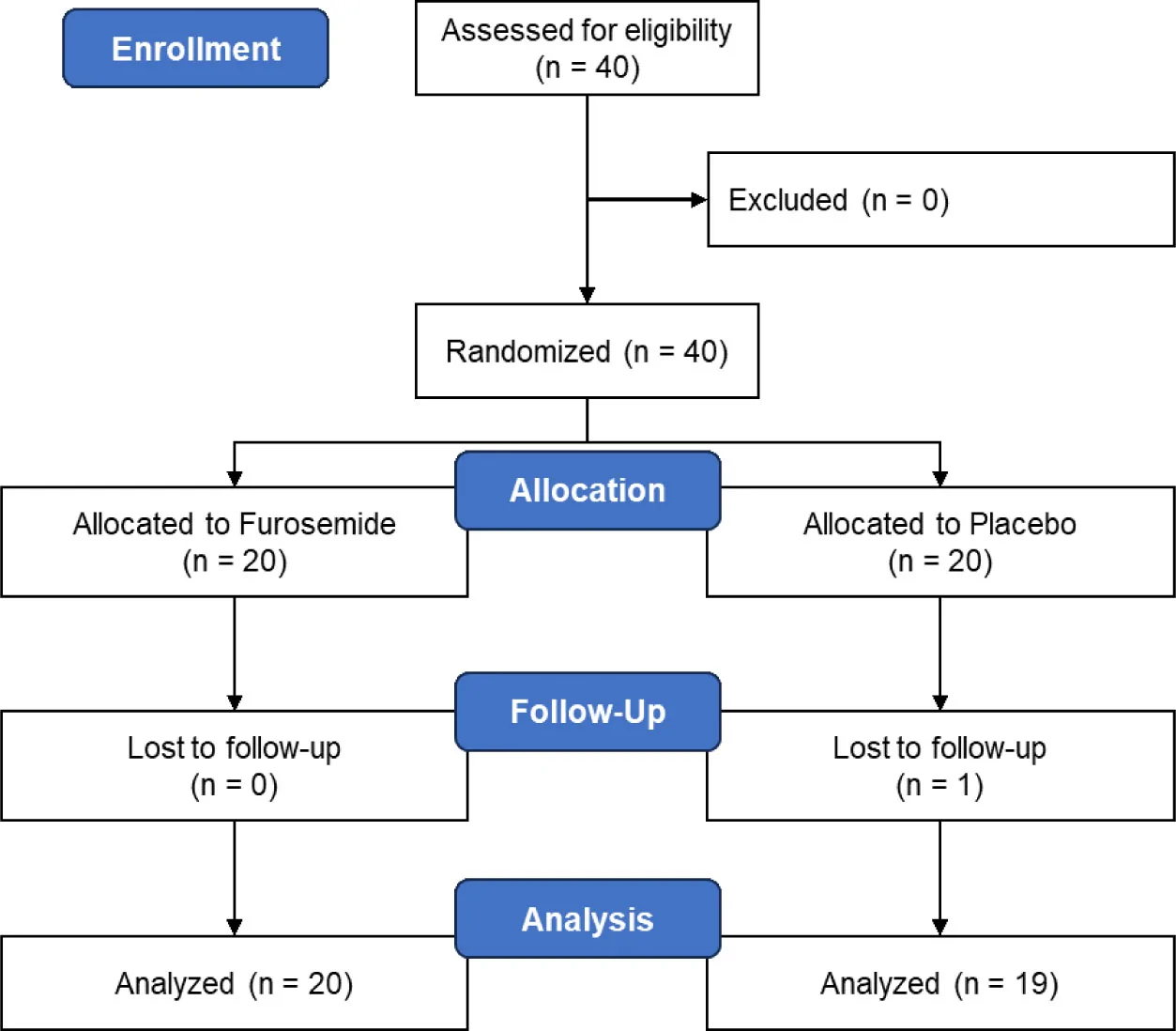
CONSORT flow diagram

Recruitment started in July 2023, and the final follow-up for the primary and secondary outcomes was completed in December 2023. The trial ended as planned after the required sample size and follow-up data were collected.

### Baseline Characteristics

The Shapiro-Wilk test revealed that age and body mass index (BMI) were non-normally distributed (P < 0.05), whereas height and weight were normally distributed (P > 0.05). The median age of the intervention group was 53.50 (IQR 18.50) years, compared to 53.00 (IQR 24.00) years in the control group, with no significant difference (P = 0.527). Gender distribution was also comparable between the groups (P = 0.421). The median BMI in the intervention group was higher (23.29 [IQR 10.71] kg/m^2^) than in the control group (19.01 [IQR 6.98] kg/m^2^), but the difference was not statistically significant (P = 0.079). Other anthropometric parameters, such as weight and height, also showed no significant differences between the groups (P > 0.05). The distribution of baseline Quick Sepsis-Related Organ Failure Assessment (qSOFA) scores (≥ 2) was higher in the intervention group (60.0%) than in the control group (40.0%). Detailed baseline characteristics are presented in Table 1.

**Table 1.** Subject characteristics

**Variable**	**Intervention (n=20)**	**Control (n=19)**	**P value**
Age (years)	53.50 (18.50)	53.00 (24.00)	0.527^a^
Gender			0.421^b^
Male	9 (45.0)	11 (55.0)	
Female	11 (57.9)	8 (42.1)	
Weight (kg)	63.50 (24.00)	52.00 (26.00)	0.066^c^
Height (cm)	160.00 (7.00)	160.00 (10.00)	0.662^c^
Body mass index (kg/m^2^)	23.29 (10.71)	19.01 (6.98)	0.079^a^
Diagnosis category			
Malignancy	7 (36.8)	12 (63.2)	
Metabolic	1 (100.0)	0 (0)	
Skin and subcutaneous infection	3 (75.0)	1 (25.0)	
Neurologic	4 (57.1)	3 (42.9)	
Respiratory	1 (50.0)	1 (50.0)	
Gastrointestinal	3 (75.0)	1 (25.0)	
Genitourinary	0 (0)	1 (100.0)	
Musculoskeletal	1 (100.0)	0 (0)	
qSOFA			0.648^b^
< 2	11 (45.8)	13 (54.2)	
≥ 2	9 (60.0)	6 (40.0)	
ICU length of stay (days)	7 (16)	6 (8)	0.607^a^
RRT usage	1 (5.0%)	1 (5.3%)	0.970^b^
28-day mortality	6 (30.0%)	7 (36.8%)	0.651^b^
APACHE II score			0.821^a^
0–4	1 (5)	1 (5.3)	
5–10	8 (40)	9 (47.4)	
11–15	7 (35)	4 (21.1)	
16–20	3 (15)	2 (10.5)	
21–25	1 (5)	3 (15.8)	
26–30	0 (0)	0 (0)	
>30	0 (0)	0 (0)	

Categorical data are presented in n (%)

Numeric data are presented in median (interquartile range)

qSOFA = Quick Sepsis Related Organ Failure Assessment; RRT = Renal Replacement Therapy; APACHE II = Acute Physiology and Chronic Health Evaluation II

a Mann-Whitney U test

b Chi-square

c Independent samples t-test

### Effect of Intervention on Fluid Distribution and BIA Parameters

Table 2 compares fluid distribution and BIA parameters among treatment arms. Cumulative fluid balance (CFB) at 0 hours was higher in the intervention group (3.41 [IQR 1.55] L) than in the control group (3.08 [IQR 3.80] L), but this difference was not statistically significant (P = 1.000). At 48 hours, CFB decreased in the intervention group by −0.34 [95% CI: −1.22–0.94] L and in the control group by −0.16 [95% CI:−1.24–1.10] L, with no significant difference observed between the groups (P = 0.863).

**Table 2.** Comparison of Fluid Distribution and BIA Parameters between Treatment Arms

**Variable**	**Intervention (n=20)**	**Control (n=19)**	**P value**
Cumulative fluid balance (L)			
Hour-0	3.41 (1.55)	3.08 (3.80)	1.000^a^
Hour-48	2.80 (4.35)	2.52 (4.13)	0.588^b^
Difference	−0.34 (2.23)	−0.16 (2.44)	0.863^b^
P value for difference	0.808^c^	0.778^c^	
Total body water (L)			
Hour-0	37.59 (9.96)	34.73 (10.14)	0.325^b^
Hour-48	35.61 (11.78)	36.30 (12.24)	0.902^b^
Difference	−1.36 (4.97)	1.51 (7.32)	0.888^a^
P value for difference	0.157^d^	0.428^d^	
Extracellular-to-intracellular water ratio			
Hour-0	0.85 (0.10)	0.87 (0.17)	0.613^a^
Hour-48	0.87 (0.19)	0.81 (0.20)	0.084^a^
Difference	0.005 (0.22)	−0.09 (0.21)	0.005^b^
P value for difference	0.332^c^	0.044^c^	
Intracellular water (L)			
Hour-0	20.60 (5.08)	17.81 (5.43)	0.087^a^
Hour-48	19.77 (7.31)	19.38 (5.01)	0.312^b^
Difference	−0.45 (3.81)	1.63 (3.66)	0.004^a^
P value for difference	0.062^c^	0.030^c^	
Extracellular water (L)			
Hour-0	17.26 (6.70)	16.85 (5.79)	0.508^b^
Hour-48	15.77 (6.19)	15.54 (3.49)	0.736^a^
Difference	−0.53 (2.64)	0.12 (2.82)	0.860^b^
P value for difference	0.550^c^	0.573^c^	
Interstitial fluid (L)			
Hour-0	13.25 (5.82)	12.70 (4.50)	0.613^b^
Hour-48	12.35 (5.45)	11.70 (2.90)	0.546^a^
Difference	−0.15 (2.38)	−0.20 (1.90)	0.475^b^
P value for difference	0.970^c^	0.409^c^	
Intravascular fluid (L)			
Hour-0	5.07 (1.35)	4.69 (1.37)	0.325^b^
Hour-48	4.81 (1.58)	4.90 (1.65)	0.900^b^
Difference	−0.19 (0.68)	0.20 (0.98)	0.116^a^
P value for difference	0.157^d^	0.422^d^	
Phase angle (°)			
Hour-0	5.30 (2.44)	6.28 (3.71)	0.055^b^
Hour-48	5.73 (3.05)	5.58 (2.88)	0.574^a^
Difference	0.34 (2.18)	−0.19 (1.77)	0.339^a^
P value for difference	0.314^c^	0.809^c^	

Data is presented in median (interquartile range)

a Mann-Whitney U test

b Independent samples t-test

c Wilcoxon signed ranks test

d Paired samples t-test

Total body water (TBW) at baseline was slightly higher in the intervention group (37.59 [IQR 9.96] L) compared to the control group (34.73 [IQR 10.14] L). At 48 hours, TBW decreased in the intervention group by −1.36 [95% CI: −2.98–1.43] L and slightly increased in the control group by 1.51 [95% CI: −2.17–2.85] L, with no significant differences between the groups at either time point or in the changes over time (P = 0.888).

The extracellular-to-intracellular water (ECW/ICW) ratio was lower at baseline in the intervention group (0.85 [IQR 0.10]) than in the control group (0.87 [IQR 0.17]), though not significantly different (P = 0.613). At 48 hours, the ECW/ICW ratio increased in the intervention group (0.87 [IQR 0.19]) and decreased in the control group (0.81 [IQR 0.20]). The difference in ECW/ICW changes between groups was statistically significant (P = 0.005), with 0.005 [95% CI: −0.03–0.16] in the intervention group and −0.09 [95% CI: −0.16–0.02] in the control group.

Intracellular water (ICW) at baseline was higher in the intervention group (20.60 [IQR 5.08] L) compared to the control group (17.81 [IQR 5.43] L), although not significantly (P = 0.087). At 48 hours, ICW in the intervention group decreased (19.77 [IQR 7.31] L), whereas it increased in the control group (19.38 [IQR 5.01] L). The changes in ICW were significantly different between groups (ΔICW = −0.45 [95% CI: −2.91–0.07] L in the intervention group vs 1.63 [95% CI: 0.71–3.29] L in the control group, P = 0.004).

Extracellular water (ECW) and interstitial fluid (IF) levels showed no significant differences between the intervention and control groups at 0 hours, 48 hours, or in their time-course changes (P > 0.05). Similarly, intravascular fluid (IVF) levels and changes over time were not significantly different between groups (P > 0.05).

Phase angle measurements at baseline were lower in the intervention group (5.30 [IQR 2.44]°) compared to the control group (6.28 [IQR 3.71]°), though not significantly (P = 0.055). At 48 hours, phase angle increased in the intervention group by 0.34 [95% CI: −0.27–1.32]° and decreased in the control group by −0.19 [95% CI: −0.70–0.63]°, but the changes were not statistically significant (P = 0.339).

Across all bioelectrical impedance analysis parameters, no significant within-group changes were observed between baseline and 48 hours, except in the control group, where the extracellular-to-intracellular water ratio (P = 0.044) and intracellular water (P = 0.030) showed statistically significant differences, indicating subtle shifts in fluid compartment distribution while other measures remained stable.

The changes in fluid distribution between the intervention and control groups are illustrated in Figure 2, and in the intervention group, TBW, ICW, ECW, IF, and IVF decreased. Conversely, in the control group, TBW and ICW levels increased, while ECW, IVF, and IF levels decreased.

**Figure 2.**
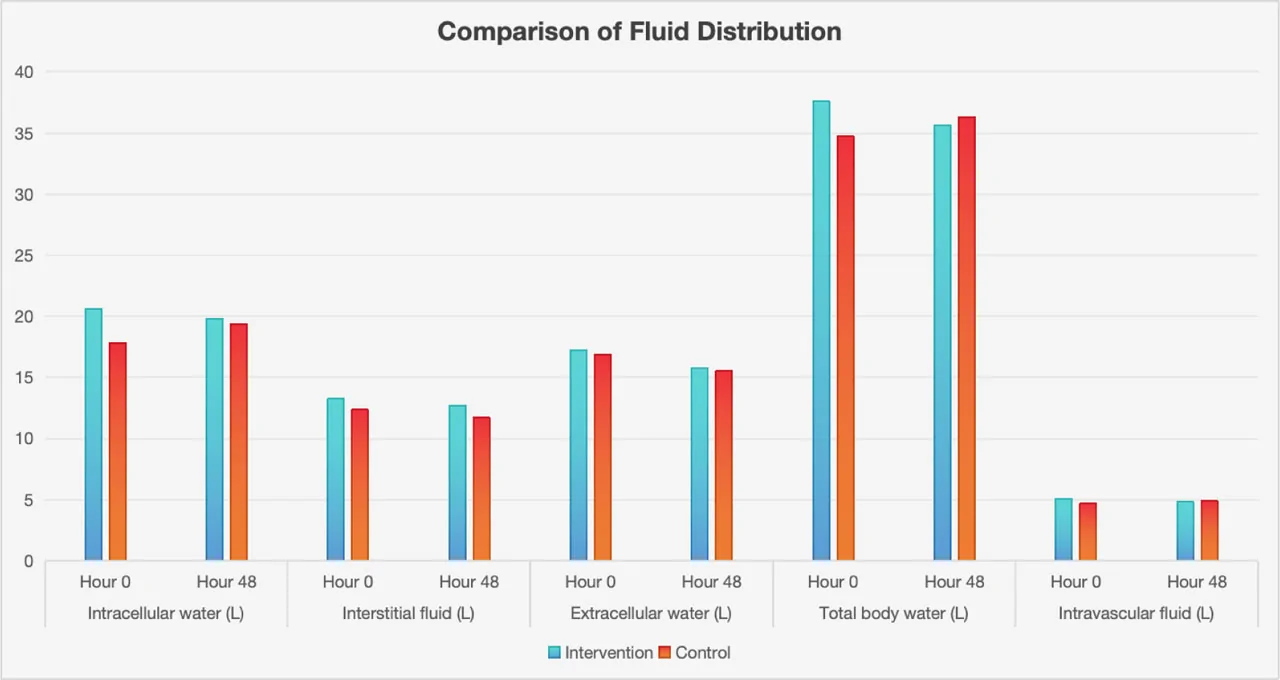
Comparison of Fluid Distribution between (a) Intervention and (b) Control Group

### Secondary Outcomes

The secondary outcomes of the study are summarised in Table 1. No significant difference was found in the ICU length of stay between the intervention and control groups (Mann-Whitney U test, P = 607). The use of Renal Replacement Therapy (RRT) was comparable between the two groups, with one patient in each group requiring it (5.0% vs 5.3%, P = 0.970).

In the analysis of mortality outcomes, the control group had a higher mortality rate than the intervention group (36.8% vs 30.0%), but this difference was not statistically significant (P = 0.651). In a subgroup analysis, no significant differences were observed in fluid distribution or BIA parameters between patients who survived and those who died (P > 0.05 for all variables). Detailed fluid distribution and BIA parameters between mortality outcomes are presented in Table 3, with a visual comparison shown in Figure 3.

**Table 3.** Comparison of Fluid Distribution and BIA Parameters between Mortality Outcomes

**Variable**	**Death (n=12)**	**Improvement (n=27)**	**P value**
Treatment Group			0.423^a^
Intervention	5 (25.0)	15 (75.0)	
Control	7 (36.8)	12 (63.2)	
Cumulative fluid balance (L)			
Hour-0	2.46 (3.22)	3.39 (1.72)	0.181^b^
Hour-48	1.59 (4.04)	3.45 (3.86)	0.373^c^
Total body water (L)			
Hour-0	37.69 (9.98)	36.34 (12.08)	0.625^c^
Hour-48	37.56 (11.33)	35.19 (11.39)	0.489^c^
Difference	−0.66 (5.11)	−0.32 (5.37)	0.692^b^
Extracellular-to-intracellular water ratio			
Hour-0	0.83 (0.14)	0.90 (0.13)	0.315^b^
Hour-48	0.81 (0.18)	0.87 (0.13)	0.153^b^
Difference	0.01 (0.18)	−0.01 (0.17)	0.771^c^
Intracellular water (L)			
Hour-0	19.71 (5.64)	19.03 (5.00)	0.584^b^
Hour-48	21.56 (8.15)	19.38 (5.31)	0.185^c^
Difference	0.11 (5.58)	0.07 (4.03)	0.855^b^
Extracellular water (L)			
Hour-0	16.41 (4.27)	17.21 (6.06)	0.882^c^
Hour-48	15.28 (3.14)	16.08 (6.29)	0.808^b^
Difference	−0.44 (2.59)	−0.15 (2.91)	0.839^c^
Interstitial fluid (L)			
Hour-0	12.30 (3.67)	13.40 (4.70)	0.802^c^
Hour-48	11.40 (1.78)	12.60 (5.20)	0.692^b^
Difference	−0.45 (2.00)	−0.10 (2.50)	0.834^c^
Intravascular fluid (L)			
Hour-0	5.09 (1.34)	4.91 (1.64)	0.624^c^
Hour-48	5.07 (1.53)	4.75 (1.53)	0.491^c^
Difference	−0.09 (0.70)	−0.04 (0.73)	0.692^b^
Phase angle (°)			
Hour-0	5.37 (4.66)	5.61 (2.15)	0.959^c^
Hour-48	5.73 (3.30)	5.58 (2.62)	0.855^b^
Difference	0.51 (4.18)	0.00 (1.38)	0.584^b^

Catergorical data is presented in n (%)

Numeric data is presented in median (interquartile range)

a Chi-square

b Mann-Whitney U test

c Independent samples t-test

**Figure 3.**
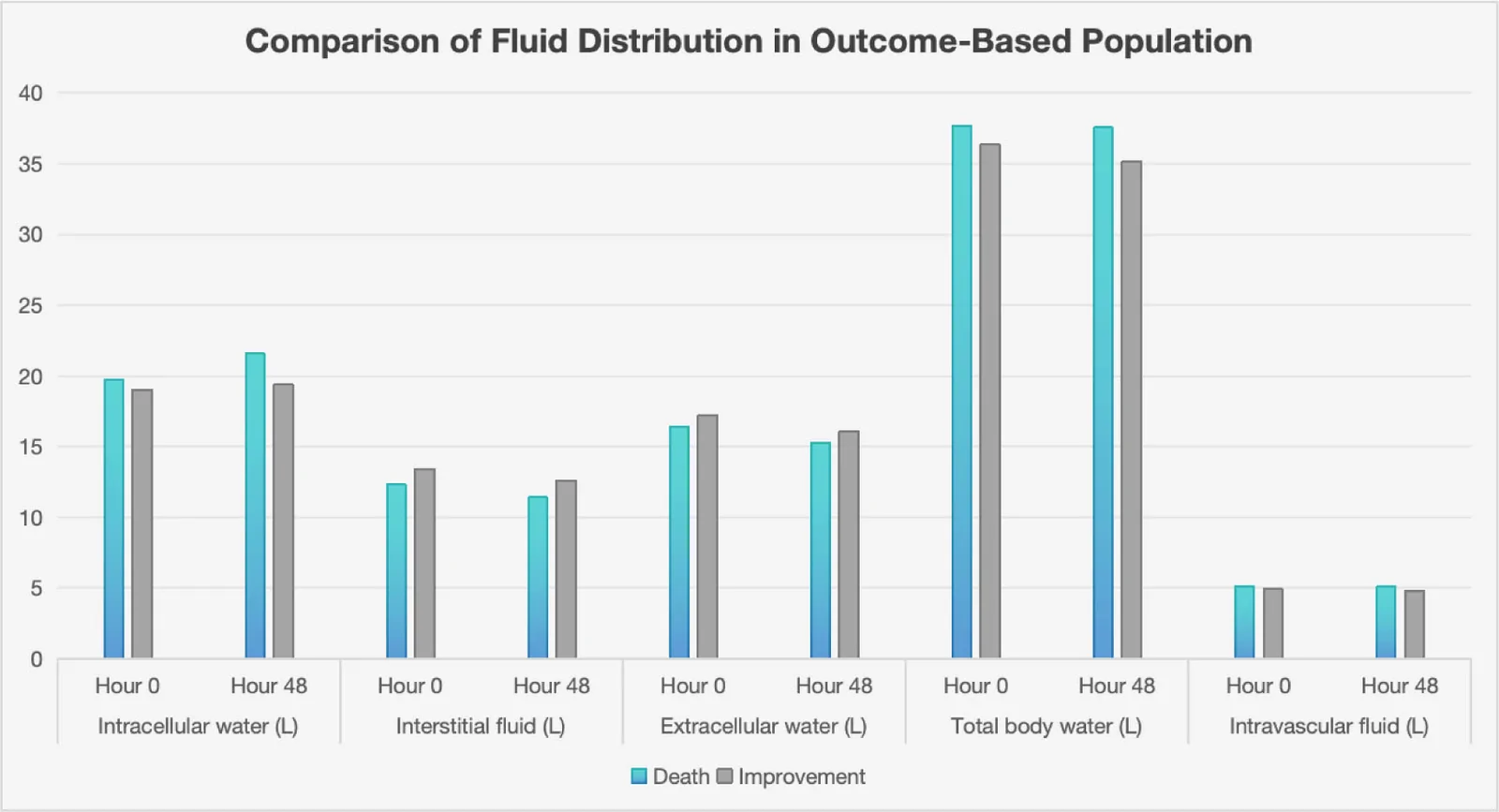
Comparison of Fluid Distribution between (a) Death and (b) Improvement Outcome

A notable observation was an increase in ICW at 48 hours among patients who died. Although this finding was not statistically significant, it is of interest because a similar pattern—an increase in ICW at 48 hours—was observed in the control group.

Table 4 compares the albumin and sodium levels between the intervention and control groups. Severe hypoalbuminemia (≤ 2.5 g/dL) was more prevalent in the control group (42.1%) compared to the intervention group (25.0%), although this difference was not statistically significant (P = 0.257). Sodium levels were categorized as low (< 135 mEq/L), normal (135–145 mEq/L), or high (> 145 mEq/L), with no significant differences between the two groups (P = 0.576).

**Table 4.** Comparison of Albumin and Sodium Levels between Treatment Arms

**Variable**	**Intervention (n=20)**	**Control (n=19)**	**P value**
Albumin level (g/dL)			0.257^a^
≤ 2.5	5 (25.0)	8 (42.1)	
> 2.5	15 (75.0)	11 (57.9)	
Sodium levels (mEq/L)			0.576^a^
Low (< 135)	6 (30.0)	5 (26.3)	
Normal (135–145)	14 (70.0)	13 (68.4)	
High (> 145)	0 (0)	1 (5.3)	

Data is presented in n (%)

a Chi square

## Discussion

This study found no significant differences in age, body weight, height, or BMI between the intervention and control groups (P > 0.05), indicating no statistically significant differences in their demographic profiles. Such homogeneity enables reliable comparison of the intervention’s effects, specifically the administration of furosemide 2 mg/hour as an early deresuscitation agent. However, the intervention group had slightly higher mean body weight and BMI, which may have clinical implications and influence outcomes due to fluid accumulation. This fluid accumulation could confound the evaluation of the nutritional status of ICU patients. These clinical characteristics could have influenced the study outcomes.

Bioelectrical impedance analysis (BIA) is a reliable, noninvasive method for assessing body fluid composition. It operates by transmitting low-level electrical currents at different frequencies through electrodes attached to the skin. High-frequency currents penetrate cell membranes, while low-frequency currents flow through extracellular fluid. The resistance to these currents, known as impedance, forms the basis of BIA measurements[8,9,19]. Fluid overload assessments using BIA rely on bioimpedance spectroscopy normalized for height, weight, gender, age, and ethnicity to calculate resistance-reactance (RXc) values, which estimate dry body weight[19]. Studies by Rochwerg et al. have validated BIA as a reliable tool for assessing fluid status in septic ICU patients, with potential benefits in avoiding excessive fluid resuscitation [20]. Kyosebekirov et al. also reported a significant correlation between BIA-derived fluid status and cumulative fluid balance[21].

Although widely used, BIA measurement is limited by multiple physiological and technical factors that can distort results. Hydration shifts, electrolyte imbalances, edema, and fluid therapy can alter impedance values. [22] Extremes of body size, obesity, or malnutrition reduce accuracy due to population-specific equations. [23] Food intake, physical activity, body temperature, posture, and electrode placement further contribute to variability, underscoring the need for standardized protocols. Even advanced multi-frequency devices face challenges in pathological states, and implants or pregnancy may affect readings without necessarily posing safety risks.[24] Overall, BIA remains safe and accessible, but its precision depends heavily on careful preparation and cautious interpretation.

Approximately 60% of the human body consists of water, distributed between intracellular and extra-cellular compartments. The extracellular fluid compartment is further divided into interstitial fluid and plasma[25,26]. In BIA, total body water (TBW) is measured alongside intracellular water (ICW), extra-cellular water (ECW), interstitial fluid (IF), and intravascular fluid (IVF)[27,28]. For critically ill patients at risk of fluid imbalance, BIA provides valuable insights into fluid status, aiding in adequate resuscitation and deresuscitation strategies. This method is particularly important for patients with acute kidney injury (AKI), who are prone to fluid imbalance [27,29].

Furosemide, a loop diuretic, facilitates fluid control and reduces renal workload in AKI patients by inhibiting the Na+/K+/Cl− cotransporter (NKCC2) in renal tubules[30]. Ruste et al. reported significant reductions in TBW, ICW, and ECW that correlated with decreased cumulative fluid balance following furosemide administration [31]. However, in this study, no statistically significant changes were observed in TBW, ICW, or ECW between baseline (0 hours) and post-intervention (48 hours). Despite this, reductions in all fluid distribution parameters were observed in the intervention group, whereas the control group showed increases in TBW and ICW. Figure 2 illustrates changes in fluid distribution between the intervention and control groups at 0 and 48 hours, revealing marked differences in TBW and ICW, although these differences were not statistically significant. The lack of significant findings may be attributable to several factors. One plausible explanation is that hypoalbuminemia, commonly observed in this population, may reduce the secretion of loop diuretics into the renal tubules, thereby reducing their effectiveness [32].

The data indicate similar median ICU lengths of stay in the intervention and control groups (7 days vs 6 days; p > 0.05). This finding aligns with the non-significant results for our other primary endpoints. Fluid overload in critically ill patients is a known contributor to prolonged mechanical ventilation and extended ICU stays. Previous research has suggested that a negative cumulative fluid balance can predict successful extubation [33], and a fluid-restrictive strategy has been shown to improve pulmonary function and shorten ICU stays compared to a liberal fluid strategy, although without a difference in mortality [34]. The lack of a significant reduction in ICU length of stay in our intervention group, despite a targeted deresuscitation approach, suggests that the regimen of continuous furosemide at 2 mg/hour may not be sufficiently potent to overcome other dominant clinical factors that determine ICU length of stay. These factors likely include the underlying reason for critical illness, as illustrated by the extreme values in our cohort (e.g., a patient with tetanus in the intervention group with a 28-day stay and a patient post-neck dissection for malignancy in the control group with a 48-day stay).

Regarding renal outcomes, some evidence suggests that diuretic therapy may help liberate patients with Acute Kidney Injury (AKI) from RRT, particularly in cases of oliguria, by promoting a negative fluid balance[35]. Furosemide has been shown to increase urine output and reduce the need for dialysis [35]. However, in our study, the rate of RRT utilization was nearly identical and not significantly different between the two groups (5.0% vs 5.3%, P = 0.970). This clearly indicates that early deresuscitation with continuous furosemide infusion was not superior to standard fluid therapy in reducing the need for RRT.

Mortality outcomes revealed no statistically significant differences between groups, though the control arm had a numerically higher proportion of deaths (36.8% vs 30.0%). Subgroup analysis indicated that deceased subjects exhibited notable increases in intracellular water, a pattern also observed in the control group. These findings should be interpreted with caution, as no definitive causal relationships can be inferred from this study.

Furosemide is expected to reduce ECW and increase ICW by promoting extracellular sodium loss[36]. Other studies have also reported that increases in ICW and decreases in the ECW/ICW ratio are markers of balanced fluid distribution and predictors of survivability [37]. However, in this study, subjects in the control group, who did not receive furosemide, exhibited increases in ICW. Severe hypoalbuminemia, which promotes plasma extravasation into interstitial and intracellular spaces, might be a hypothetical explanation behind this [38]. Table 4 shows that severe hypoalbuminemia occurred more frequently in the control group (42.1% vs 25.0%), though this difference was not statistically significant (P = 0.257). However, this finding does not align with the theoretical expectation that hypoalbuminemia would increase IF alongside ICW, as observed in this study [39].

Another factor that may potentially influence ICW increases is osmolarity disturbances. Hypoosmolar conditions facilitate rapid fluid shifts from extracellular to intracellular compartments, leading to cellular edema [26]. Hyponatremia was observed in 30.0% of the intervention group and 26.3% of the control group. However, osmolarity was not measured in this study, limiting the ability to ascertain its contribution to fluid shifts and mortality. The proposed hypothesized pathophysiological mechanism of osmolarity disturbance in this study may be attributable to overhydration in subjects not undergoing furosemide deresuscitation. As shown in a study by Rodríguez-Moguel et al., overhydration increased ICW/TBW ratios and decreased albumin levels, correlating with higher mortality rates (OR: 2.82, 95% CI: 1.05–7.50, P = 0.03)[40]. This underscores the importance of fluid deresuscitation in critically ill patients and challenges the assumption that a lower ECW/ICW ratio predicts better outcomes.

### Limitations of the Study

Several limitations must be considered when interpreting the results of this study. First, the relatively small sample size of 39 patients limits the statistical power of our findings. The short duration of the study, with outcomes assessed only at 48 hours, is another limitation. While this timeframe provided insight into immediate fluid redistribution and some early physiological effects, it may not be sufficient to capture the long-term consequences of the intervention on patient outcomes, including recovery and mortality. Furthermore, osmolarity was not measured in this study, limiting our ability to assess its potential role in fluid shifts and the pathophysiology of cellular edema. Another limitation lies in the use of bioelectrical impedance analysis (BIA) to measure fluid distribution. While BIA is a well-established, noninvasive method, factors such as electrode placement, body temperature, and hydration status can affect its accuracy. These variables may have introduced measurement error, thereby affecting the reliability of the fluid distribution data.

These limitations highlight the need for further research with larger, adequately powered trials, more extended follow-up periods, and additional measurements, such as osmolarity, to provide a clearer understanding of the role of furosemide in fluid management and its impact on outcomes in critically ill patients.

## Conclusion

Furosemide-induced deresuscitation appears to influence fluid redistribution between the intracellular and extracellular compartments, though its effect on clinical outcomes remains inconclusive. Hypoalbuminemia may attenuate furosemide’s efficacy, highlighting the importance of addressing albumin status in fluid management strategies for critically ill patients. Further research is needed to clarify the relationship between fluid dynamics and clinical outcomes in this population.

## Supplementary Material

Supplementary Material Details
